# Reliability of suprapubic pedicled phalloplasty to address genital gender‐affirming surgery in transgender men: A single‐center cohort analysis

**DOI:** 10.1111/andr.70074

**Published:** 2025-05-27

**Authors:** Mattia Anfosso, Lorenzo Cirigliano, Mirko Preto, Paolo Gontero, Marco Falcone

**Affiliations:** ^1^ Center for Reproductive Medicine and Andrology/Clinical and Surgical Andrology University Hospital of Münster Münster Germany; ^2^ Urology Clinic ‐ A.O.U. “Cittàdella Salute e della Scienza”‐ Molinette Hospital University of Turin Turin Italy; ^3^ Neurourology Clinic ‐ A.O.U. “Città della Salute e della Scienza” – Unità Spinale Unipolare Turin Italy; ^4^ Urology Department Medical Faculty Biruni University Istanbul Turkey

**Keywords:** phalloplasty, total phallic construction (TPC), transgender men

## Abstract

**Background:**

Over the years, numerous techniques have been used to address genital gender‐affirming surgery (GGAS) in transgender men. Among the proposed surgical options to address TPC in transgender men, suprapubic pedicled phalloplasty (SPP) has rarely been considered in the current scientific literature.

**Objectives:**

The aim of the study is to report the surgical outcomes of the first step of suprapubic pedicled phalloplasty (SPP)—with or without urethral lengthening (UL)—evaluating possible risk factors affecting the incidence of complications.

**Methods:**

The study was conducted as a retrospective, single‐center analysis at a regional tertiary referral center. Between April 2006 and August 2024, 40 transgender men underwent GGAS at our center. GGAS was conducted as a multistage procedure, consisting of (1) SPP (2) eventual radial artery‐based forearm free‐flap urethroplasty, (3) glans sculpting, join‐up, vaginectomy and scrotoplasty, and (4) penile prosthesis implantation. Surgery time, intra‐ and postoperative complications, and hospital stay were selected as variables for surgical outcomes.

**Results:**

The median operative time was 130 min (111–158 min). Partial necrosis of the phallus was detected in 4 cases (10.5%) it was easily managed through a minor outpatient procedure. The median length loss after debridement was 1.3 cm (0.5–2 cm), without significant impact on the final length. A total loss of the neophallus occurred in a single case (2.5%) and required a staged salvage TPC. In the present series, only 14 patients (35%) opted for urethral reconstruction: 8 (20%) required a perineostomy, 4 (10%) underwent radial artery urethroplasty (RAU), while the remaining 5% required an additional metoidioplasty with urethral lengthening, ensuring that the clitoris was not incorporated into the neophallus during penile construction. Univariate and multivariate analyses failed to highlight any possible risk factors influencing the incidence of postoperative complications. The lack of a comparison group or randomization, the limited follow‐up, and the absence of patient‐reported outcome analysis are the main limitations of our study.

**Conclusion:**

Our evidence suggests that SPP is a reliable and technically accessible option for GGAS in transgender men, particularly when microsurgery is contraindicated. The technique offers the advantage of accommodating the patient's needs for urethral lengthening, simplifying the surgical process, and reducing operative times. While vascular complications may occur relatively frequently, they are mostly minor and can be managed with simple outpatient procedures.

## 1 INTRODUCTION

Genital gender‐affirming surgery (GGAS) has always been considered in all its forms one of the most challenging areas for surgeons. Total phallic construction (TPC) represents the most difficult phase in the genital gender affirmation pathway, aiming to achieve aesthetic and functional outcomes of an anatomical structure that to date cannot be perfectly constructed. Therefore, phalloplasty is univocally considered a surgical challenge. An ideal phalloplasty should include a cosmetically acceptable appearance, both tactile and erogenous sensation, an incorporated neourethra to enable urination while standing from the tip of the neophallus, adequate bulk to facilitate the insertion of an internal stiffener to guarantee the rigidity necessary for penetrative sexual intercourse.^1^, ^2^, ^3^

Meeting most of these criteria, in the lack of any comparative study highlighting a widely recognized gold standard approach, radial forearm artery free flap phalloplasty (RAFFF) is currently considered to be one of the preferred techniques among the several options on offer.^4^, ^5^, ^6^, ^7^

However, the high incidence of intra‐ and postoperative complications described in the literature, such as strictures and fistulae of the neo‐urethra,^8^, ^9^ visible and stigmatizing scars^10^ as well as sensitivity disorders at the donor site,^11^ and partial or total loss of the neo‐phallus,^12^ may make this option less attractive and desirable to the patient. Careful selection based on known risk factors like smoking habit,^13^, ^14^, ^15^ diabetes mellitus or BMI,^16^ and morbidity of the donor site also make many patients noneligibly for this type of technique, thus forcing the search for alternatives.

Today, there is clearly no technique that can compensate for all the above‐mentioned disadvantages of RAFFF, but it is possible, depending on the different needs of the patient and according to WPATH standards of care,^17^ to find valid alternatives.

Among the proposed surgical options to address TPC in transgender men, the suprapubic pedicled phalloplasty (SPP), following the original report by Bettocchi et al.,^18^, ^19^ has rarely been considered in the current scientific literature. It relies on a pedicled abdominal fascio‐cutaneous flap and it is featured by its vascular reliability and concealable low abdominal scar.^19^ The resulting neophallus lacks a neourethra, which can be provided at a later stage either with grafts or rather with a urethroplasty with a free radial artery flap (RAU),^17^, ^20^ if required.

The decision to perform an SPP can be based on several factors:

1. Willingness to undergo a safe and versatile technique, particularly suitable for patients who are not interested in upright urination or who prefer to undergo urethral lengthening (UL) at a later stage.
2. Availability of suprapubic/abdominal adipose tissue appropriate for performing the technique.
3. In cases where urethral lengthening is not required, SPP is a possible option for patients with vascular risk factors who are not eligible for a RAFFF.
4. In case UL is required, and in patients without cardiovascular risk factors, this stepwise approach appears to reduce microsurgical complications, aligning with recent international trends.^21^, ^22^, ^23^, ^24^ The interval between surgical stages allows for vascular autonomization^25^, ^26^, ^27^ while maintaining a complication rate comparable to free flaps but with lower donor site morbidity,^23^, ^28^ as the skin pad required for neourethral construction is more limited in SPP combined with RAU than in a RAFFF.

However, there are some limitations to consider. Aesthetically, the shape of the neophallus is not cylindrical but rather pyramidal, with a tendency to widen at the base, and a permanent dorsal scar remains visible. Functionally, the absence of nerve anastomoses results in little to no penile sensitivity. Additionally, the need for a second surgical step may cause further distress for the patient. Finally, this technique cannot be performed on patients without a sufficient abdominal adipose layer or, in the case of desired urethral lengthening, on those with cardiovascular risk factors.

The aim of our study is to report the surgical outcomes of a consecutive series of transgender men who underwent SPP as the first step of the GGAS path in a single tertiary referral center.

## 2 MATERIALS AND METHODS

A retrospective analysis of transgender men who underwent SPP was conducted by extrapolating data from medical records and operative notes. An independent and blinded double review of all data was performed by MA and LC to avoid oversight and bias.

All patients underwent a structured diagnostic and therapeutic pathway according to WPATH standards of care.^17^ All patients were evaluated by a multidisciplinary team of clinicians, which included psychiatrists, psychologists, endocrinologists, and surgeons. Finally, we recruited transgender men referred to our center to undergo a TPC as the last step of their gender‐affirming path.

We set as exclusion criteria:

- Cis‐gender men requiring a TPC
- Patients unwilling to participate in the study
- Patients lost at follow‐up

A descriptive analysis of patients’ features and surgical outcomes of SPP was reported. Intraoperative and postoperative complications were described and classified according to the Clavien–Dindo classification.^29^ Patients were evaluated once a week for the first month, then once every 3 months during the first year, and finally once a year. The postoperative complications listed refer to the first major surgical step and are to be understood as early, as they all arose in the 30‐day period after surgery. Long‐term complications, as in the case of urethral outcomes, are not reported in this article.

Specifically, we assessed the following complications:

- Hematoma: defined as localized, self‐limiting bleeding, not requiring active intervention, observed at the mid/low abdominal suprafascial or genital level.
- Bleeding: blood loss requiring transfusion.
- Infection: redness, swelling, and suppuration at any level of the surgical wound, requiring advanced wound dressing and antibiotic therapy.
- Partial necrosis: localized, self‐limiting necrosis not involving the entire neophallus, usually located distally and requiring surgical debridement.
- Total loss of the flap.

Predicting factors for postoperative complications were analyzed. All patients obtained written informed consent to undergo GGAS and to eventually publish images of the surgical procedure. The study was approved by the local ethical committee (Prot. N° 0079419 del 26/07/2021).

### 2.1 Surgical technique

**FIGURE 1:**
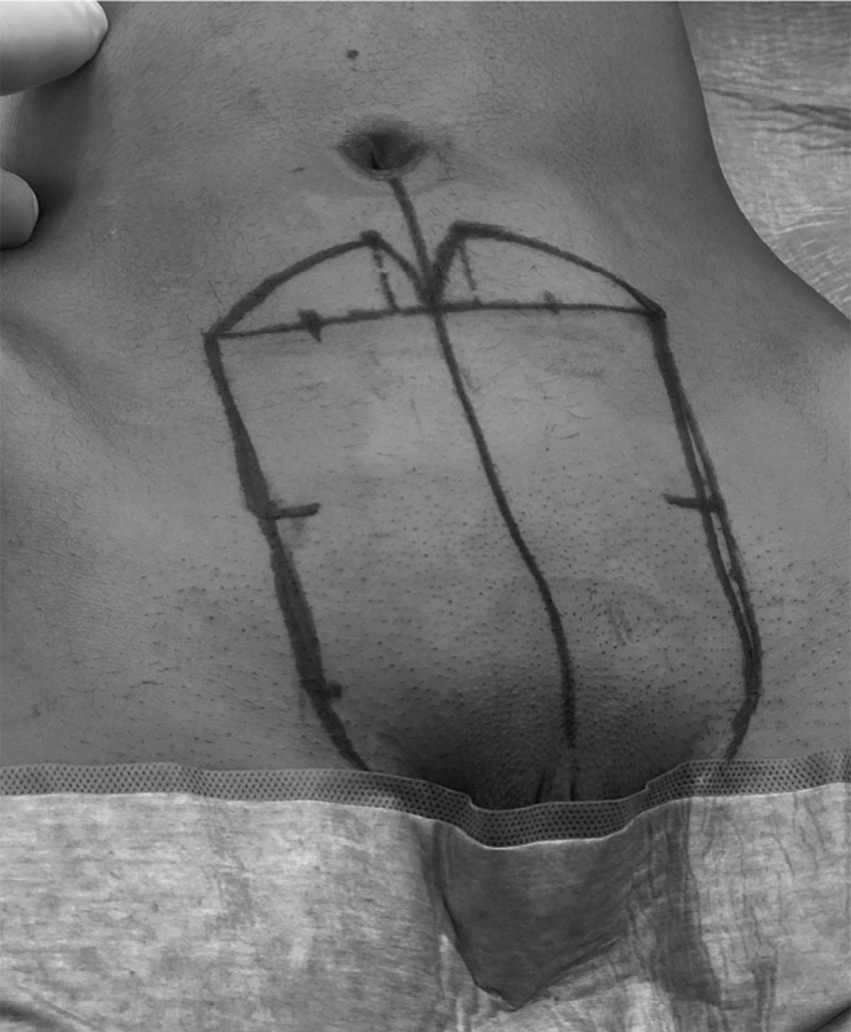
Flap design.

**FIGURE 2:**
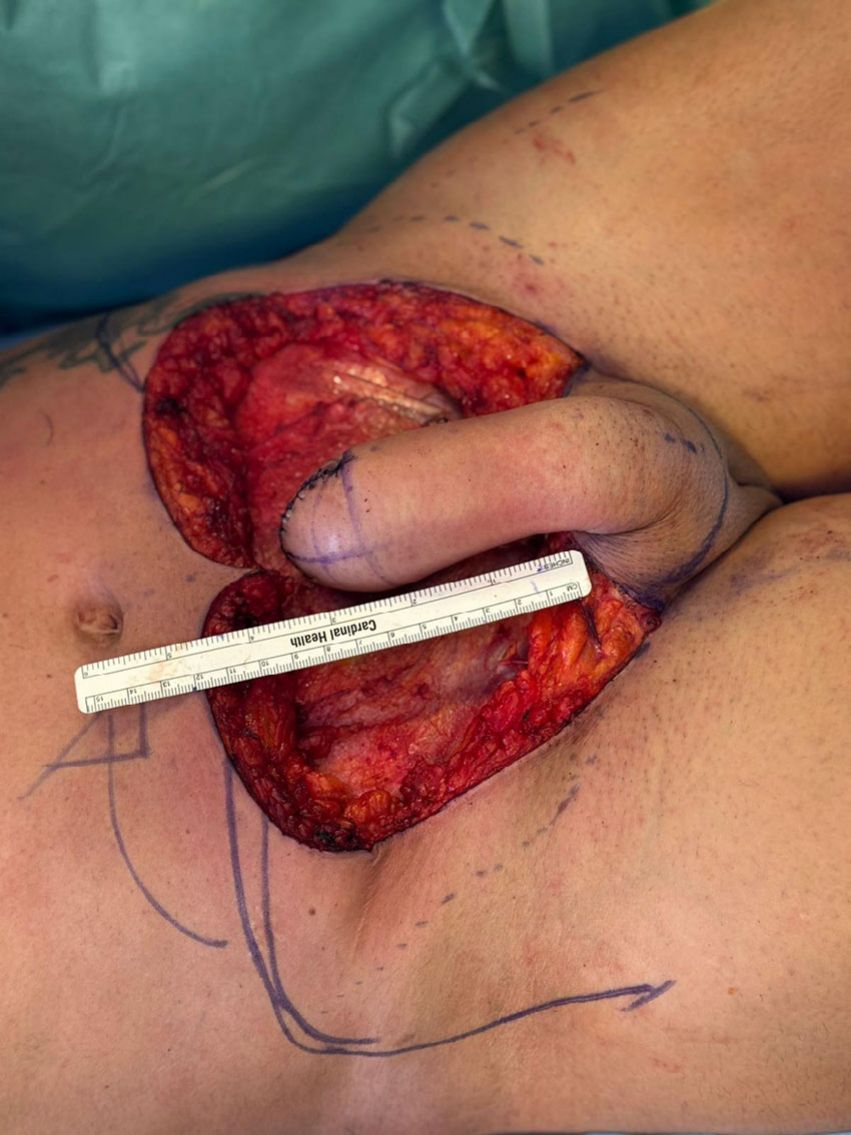
Raising of the flap and construction of the neo‐phallus.

**FIGURE 3:**
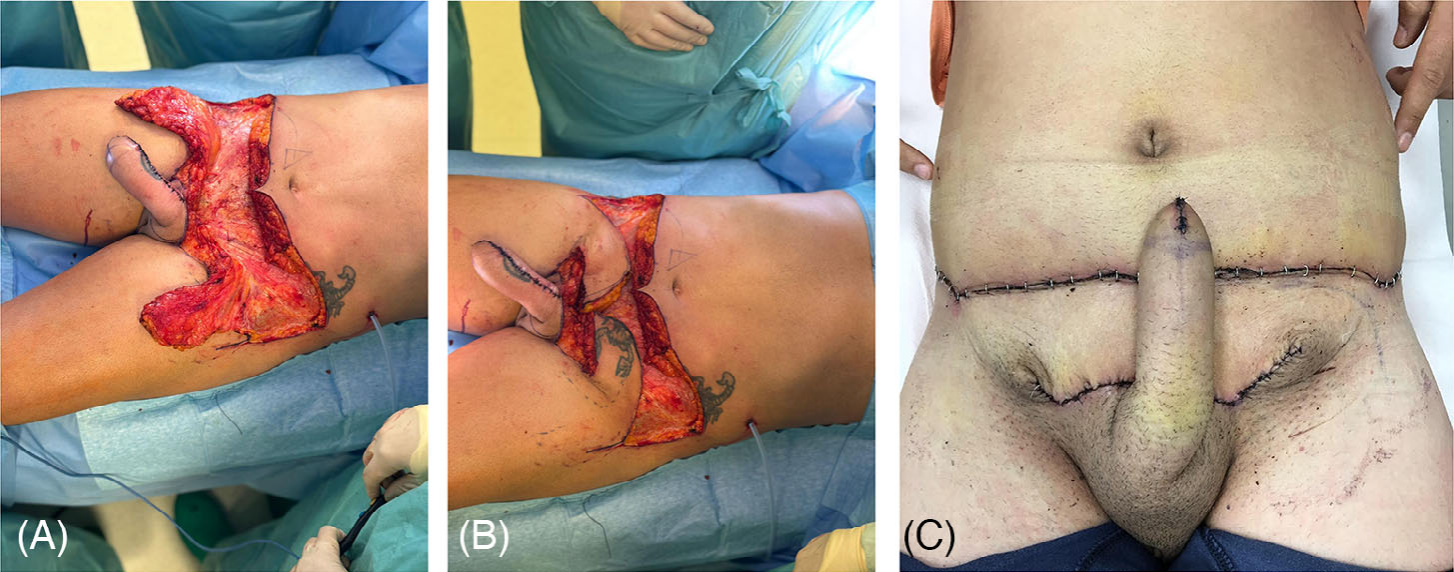
(A, B) Primary closure of the abdominal defect with two rotational pedicled aps. (C) Final result of SPP step 1.

**FIGURE 4:**
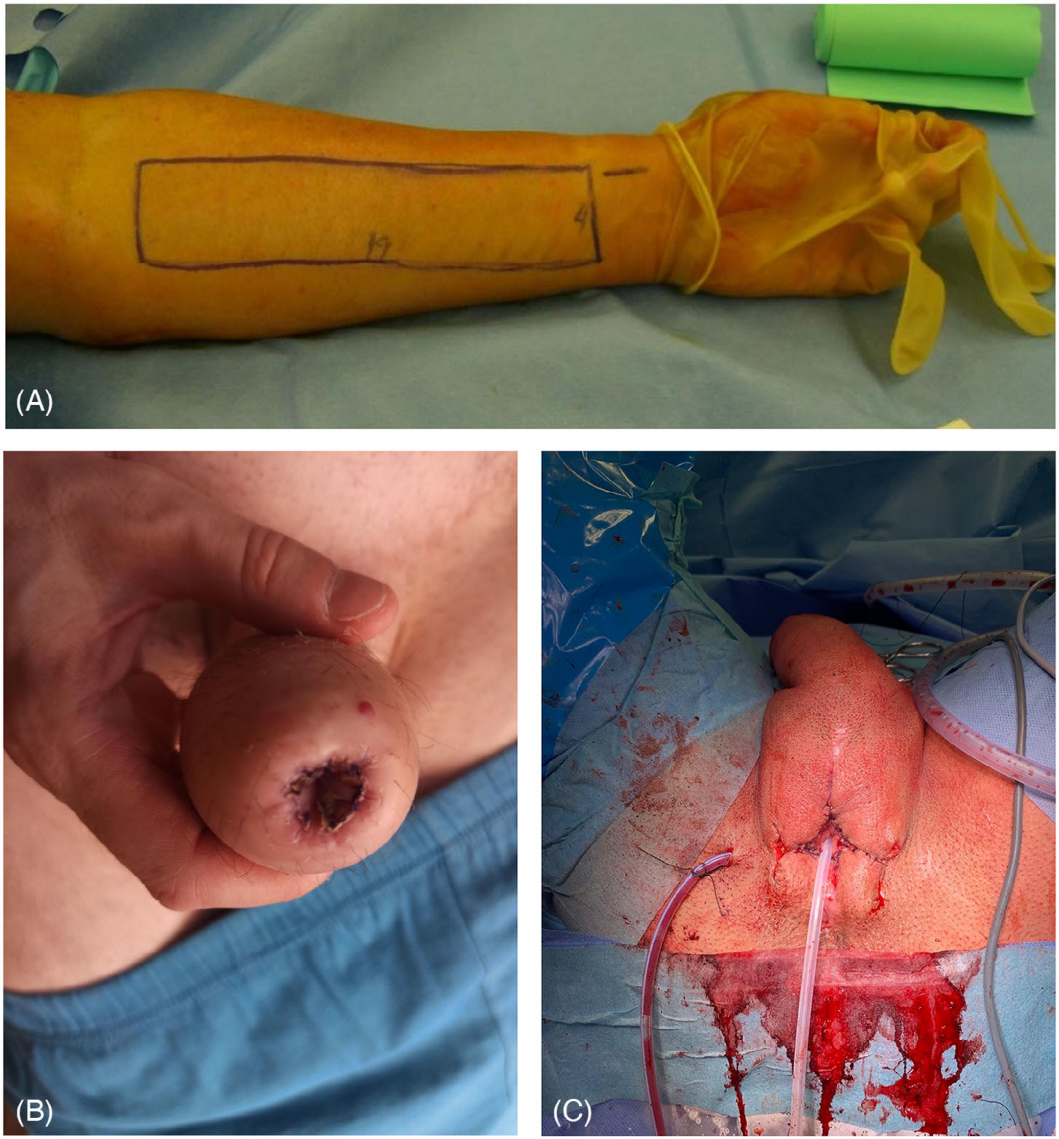
(A) Radial artery‐based free forearm urethroplasty (RAU) design. (B) Incorporation of a radial artery‐based free forearm urethroplasty (RAU). (C) Urethral advancement, vaginectomy, and perineostomy configuration.

**FIGURE 5:**
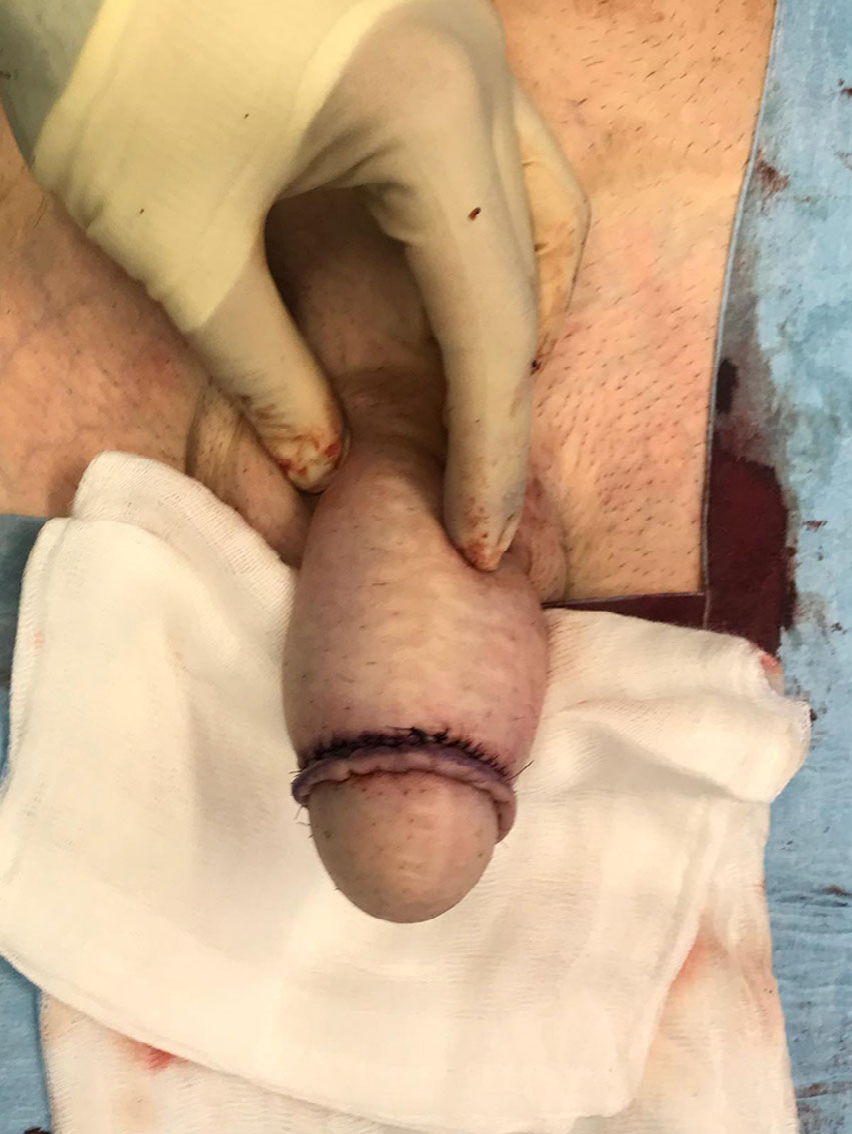
Neo‐glans sculpting using a modified Norfolk technique.

**FIGURE 6:**
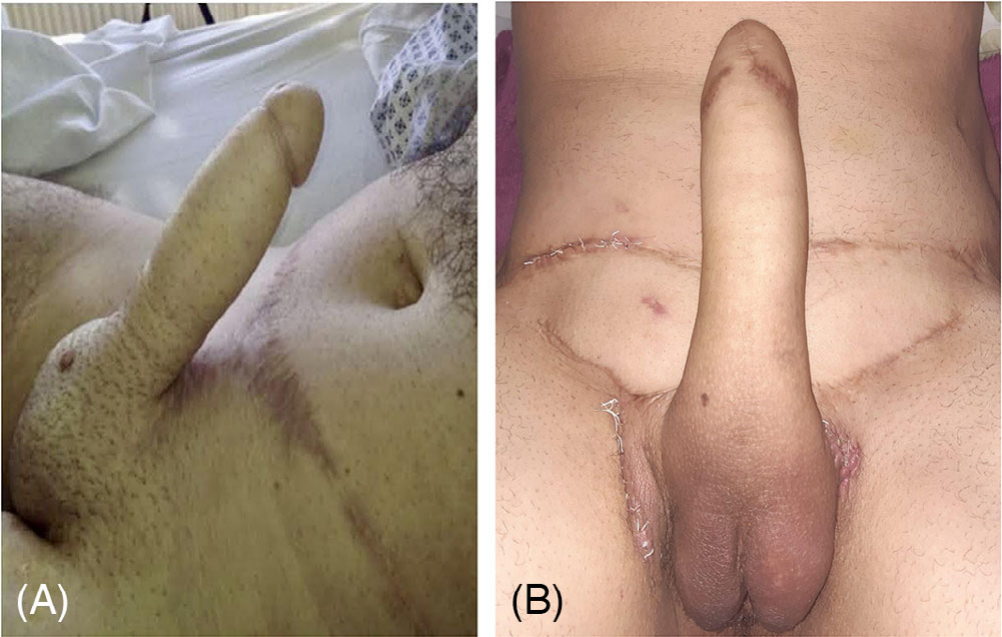
(A, B) Final results after penile prosthesis implantation.

In all cases, GGAS was performed as a staged procedure by the same experienced surgeon:

1. SPP to address TPC (Figures 1, 2, 3)
2. Urethral reconstruction—if required—which was addressed using three surgical approaches:
3. Incorporation of a radial artery‐based free forearm urethroplasty (RAU), (Figure 4A,B)
4. Metoidioplasty^30^
5. Urethral advancement, vaginectomy and perineostomy configuration^31^, ^32^ (Figure 4C)
6. Glans sculpting and urethral join‐up, vaginectomy, and scrotoplasty in case of previous RAU (Figure 5)
7. Penile prosthesis implantation^33^, ^34^ (Figure 6A,B)

**FIGURE 7:**
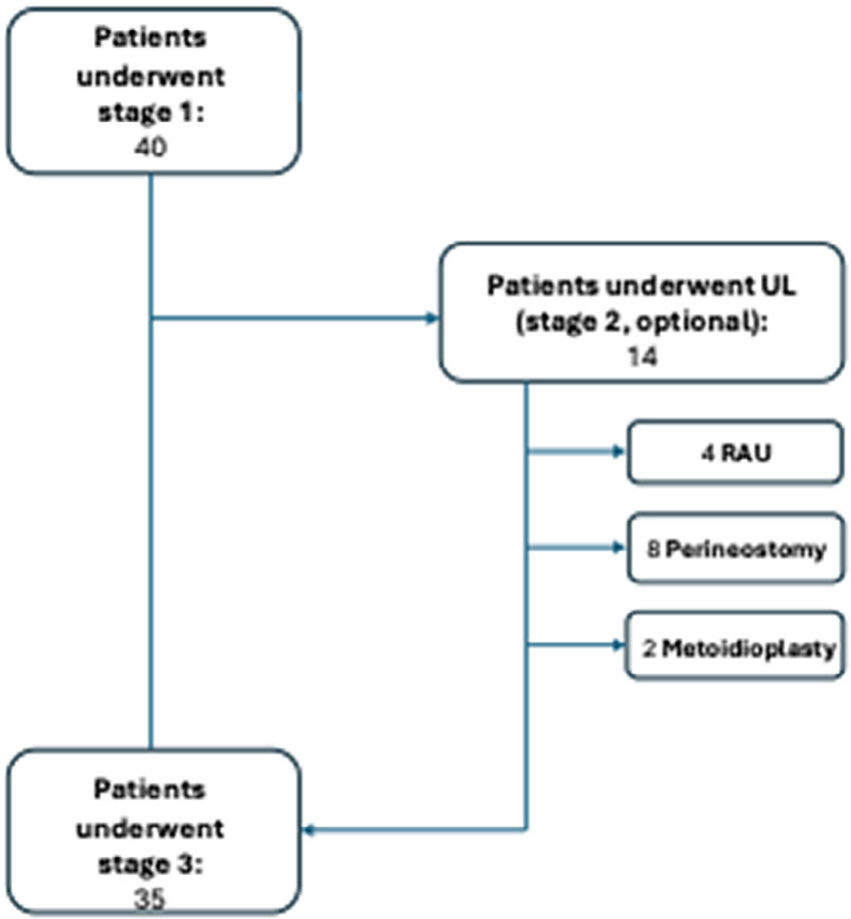
Flow chart of the operational steps. All patients underwent neo‐glandoplasty using the Norfolk technique in accordance with their aesthetic preferences.

The following paragraph reports a step‐by‐step description of the SPP technique. Figure 7 provides a flow chart describing the number of patients undergoing each operational step.

The procedure was performed under general anesthesia. An indwelling 16 Ch bladder catheter was placed. The patient was positioned in the lithotomy position.

A suprapubic flap measuring 12 cm in width and 13 cm in length, starting from the base of the clitoris, was marked (Figure 1). The flap was progressively dissected from the sheath of the rectus abdominis muscle. Extreme care was taken during the dissection of the proximal area of the flap to preserve the superficial external pudendal vessels, the inferior epigastric artery and vein, and the genitofemoral nerve emerging from the external inguinal orifice, which must be incorporated into the base of the flap bilaterally. After full‐mobilization of the flap was obtained, the excess subcutaneous fat was carefully excised exposing Scarpa's fascia to enable a tension‐free tabularization of the neophallus. At the end of the neophallus creation a consistent abdominal defect was created (Figure 2). A cranial abdominal fascio‐cutaneous flap was mobilized to facilitate the closure of the abdominal defect. In the case of extended abdominal flap dissection, to reduce skin tension and avoid the risk of umbilical necrosis, the umbilical scar is incised and the umbilicus is repositioned caudally. If the abdominal advancement flap was deemed to be insufficient to achieve a tension‐free primary closure of the abdominal defect, two rotational pedicled flaps based on the external circumflex artery were isolated to assist in the closure (Figure 3A–C).

Despite the aforementioned urethral reconstructive options, a neoglans was created in all cases following the patient's desire, mobilizing a circumferential coronal flap and applying a full‐thickness skin graft raised from the right iliac spine to create the coronal sulcus. This procedure was performed at least 3 months after the TPC to prevent any possible vascular complications (Figure 5).

Penile prosthesis implantation was carried out at least 1 year after the TPC, once all potential urethral complications had been addressed (Figure 6A,B). The type of implant inserted may include either semi‐rigid or inflatable devices. The number of cylinders implanted is generally planned in a preoperative setting, according to the bulk of the phallus. The surgical steps of this procedure follow those previously described in a recent publication.^3^

Patients were discharged with prophylactic oral antibiotics for 1 week and were reviewed to exclude early postoperative complications.

### 2.2 Statistical analysis

The normality of the variables’ distribution was tested using the Shapiro's test. Categorical variables were described using frequency and percentage, and continuous variables were described using the median and interquartile range (IQR for nonnormally distributed variables). Univariate logistic regression was performed to identify predictive factors for postoperative complications. Two‐sided *p* < 0.05 was set to be statistically significant. Statistical analysis was carried out using R (version 2024.04.2+764).

## 3 RESULTS

Between April 2006 and August 2024, a consecutive series of 45 transgender men underwent GGAS at our center. Forty of them met the inclusion criteria and were therefore included in the present study. Patient characteristics are summarized in Table 1. The mean age was 38.9 years (SD 9.91). The median BMI was 23.9 (IQR 22.9–27). Median follow‐up was 12 months (IQR 2–24.5). Operatives’ features are summarized in Table 2. The median operative time was 130 min (IQR 111–158). A defatting of the flap was necessary in 35 patients (87.5%) as well as a caudal repositioning of the umbilicus in 24 patients (60%). Lateral rotational flaps to close the abdominal defect were required in 27 patients (67.5%), while a direct closure of the abdominal defect was possible in the remainder. Median flap measurements were: 13 cm in length (IQR 12–14) and 12 cm in width (IQR 11–12). The mean hospital stay was 6.4 days (SD 1.7).

**TABLE 1:** Descriptive feature of a series of 40 transgender men who underwent suprapubic peddled phalloplasty.

Variables	Total
Number of patients, *n*	40
Mean age (SD), year	38.9 (9.91)
BMI (IQR), value	23.9 (22.9–27)
Smoking habit, *n* (%)	12 (30.8)
Diabetes, *n* (%)	1 (2.5)
Previous abdominal scar, *n* (%)	1 (2.5)

**TABLE 2:** Intraoperative and postoperative outcomes at 1‐year follow‐up of 40 transgender men who underwent suprapubic pedicled phalloplasty.

Operative time (IQR), min	130 (111–158)
Mean hospital stay, (SD) days	6.4 (1.7)
Flap defatting, *n* (%)	35 (87.5)
Umbilicus caudal repositioning, *n* (%)	24 (60)
Lateral rotational flap, *n* (%)	27 (67.5)
Length of the flap (IQR), cm	13 (12–14)
Width of the flap (IQR), cm	12 (12–12)
Intraoperative complications, *n* (%)	1 (2.5)
Postoperative complications, *n* (%)	11 (27.5)
Partial necrosis of the flap, *n* (%)	4 (10.5)
Total loss of the flap, *n* (%)	1(2.5)
Wound infection, *n* (%)	2 (5)
Haematoma / Bleeding, *n* (%)	5 (12.5)
Clavien Dindo 1, *n* (%)	2 (5)
Clavien Dindo 2, *n* (%)	3 (7.5)
Clavien Dindo 3b, *n* (%)	4 (10)
Staged urethral reconstruction, *n* (%)	14 (35)
Radial artery urethroplasty, *n* (%)	4 (10)
Perineostomy, *n* (%)	8 (20)
Metoidioplasty, *n* (%)	2 (5)

In the present series, only 14 patients (35%) desired a urethral reconstruction: 2 (5%) required a metoidioplasty, 8 (20%) a perineostomy, whereas an RAU was performed in the remainder (10%).

**FIGURE 8:**
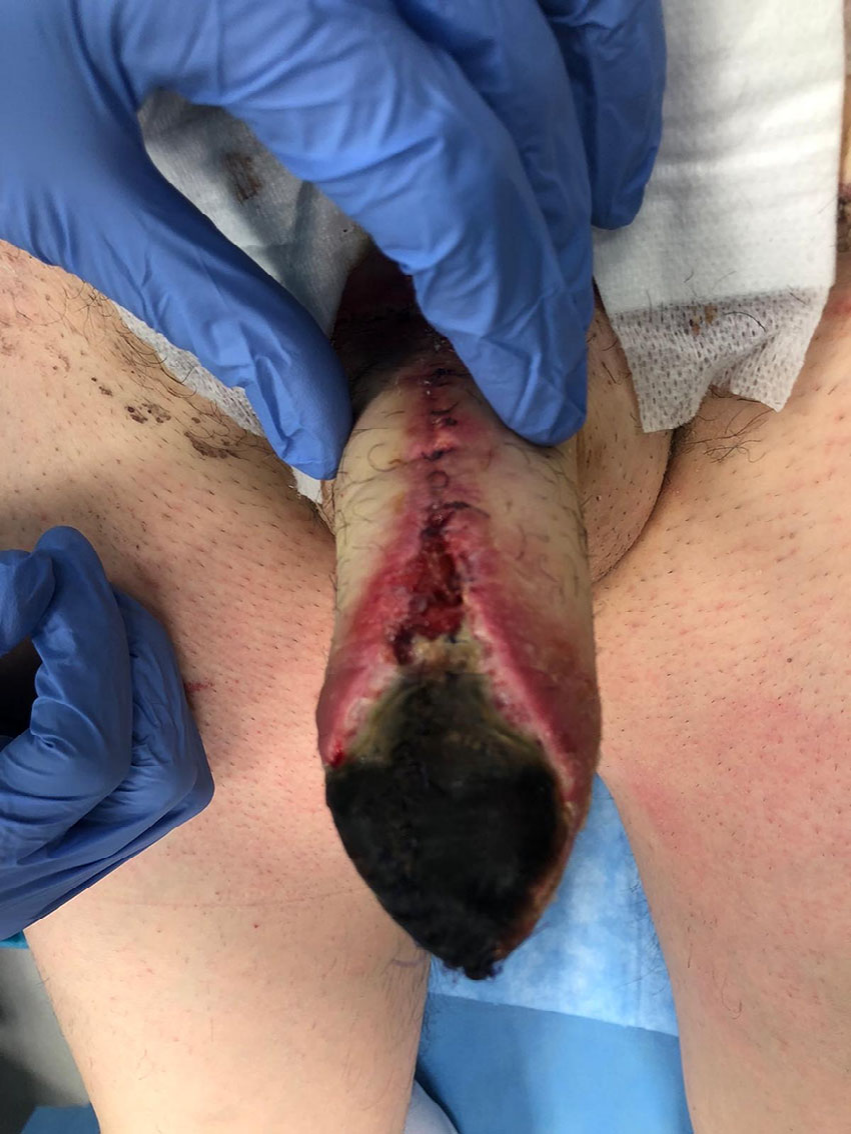
Partial distal necrosis of the phallus.

**FIGURE 9:**
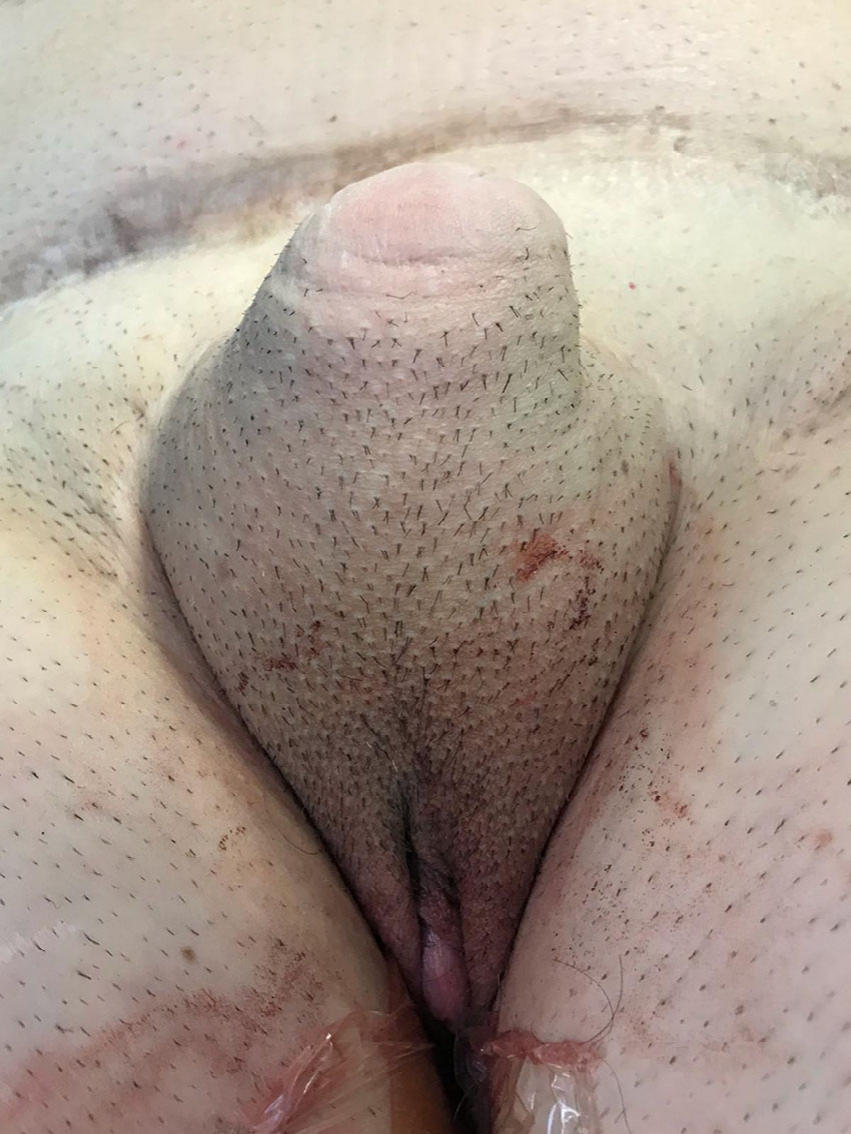
Penile stump after total loss of the neophallus.

Overall, the incidence of postoperative complications was low, confirming the reliability of this flap in addressing TPC. Two cases of wound infection (5%) (grade 2) and 5 cases of hematoma (12.5%) (grade 2) were observed. A partial necrosis of the phallus (Grade 3) was detected in 4 cases (10.5%, Figure 8). Being localized in the distal phallus in all cases due to the vascular anatomy of the flap, it was easily managed through a minor outpatient procedure involving debridement and neophallus tip reconfiguration, performed without the need for anesthesia. Median length loss after debridement was 1.3 cm (0.5–2), which did not have a great impact on the length of the neo‐phallus. A total loss of the neophallus occurred in a single case (2.5%)—(grade 3, Figure 9) and it required a staged salvage TPC.

None of the analyzed variables (smoking, diabetes, BMI, operative time, number of operative stages, and defatting) were statistically linked to the incidence or type of postoperative complications in univariate logistic regression analysis. The same result was confirmed by multivariate logistic regression analysis.

## 4 DISCUSSION

Since its first description in 1936 by Bogoras,^35^ TPC has undergone numerous modifications and evolutions over time. The advent of new technologies, together with the variety of patient needs and the current impossibility to fully replicate the complex anatomy and physiology of the penile structure has led to the development of a wide range of techniques.

Currently, the available techniques are: (1) the RAFFF,^4^ (2) the SPP,^36^ (3) the anterolateral thigh flap,^37^, ^38^ which can be performed either as a pedicled or free flap, (4) the latissimus dorsi flap,^39^, ^40^ which is performed as a free flap, (5) the superficial circumflex iliac artery perforator flap (SCIP)^22^, ^41^ and (6) the scapular flap.^42^, ^43^ Last but not least, metoidioplasty^44^, ^45^ which, although it cannot be considered a full TPC, remains one of the options for genital construction in this category of patients.

Ideally, according to the criteria listed by Hage J. et al.,^1^, ^2^ the goal of a TPC would be to recreate an aesthetically acceptable penis, with sufficient mass to allow the insertion of a penile prosthesis and with excellent functional results in terms of urination and penetration capacity. Placing these criteria as the ultimate goal of TPC, the RAFFF may be considered the best option as it allows the construction of a sensate neophallus with a neourethra in a single‐stage fashion. As described in the literature,^6^, ^20^, ^46^ this procedure guarantees satisfactory anatomical and functional results, offering the patient a neophallus of a decent size, suitable for standing urination and penetration when armed with a penile prosthesis implantation. Nevertheless, the complexity of the procedure, the incidence of complications, and the visibility of the donor site are inevitably accompanied by a high amount of stress for the patient, both physical and psychological, which becomes chronic when we consider the stigmatizing and barely hideable scars left on the forearm.^7^, ^47^, ^48^ Finally, single‐stage urethral reconstruction is associated with a high rate of postoperative complications such as stricture and fistula, ranging from 32% to 58%, with possible multiple revisions required.^8^, ^9^, ^49^, ^50^, ^51^

It is therefore understood that a true gold standard to address TPC has not yet been reached. However, by shifting the focus from Hages’ ideal criteria to the concrete needs of patients, we can find viable alternatives.^52^ Among these options, SPP may represent a valuable option for patients with a decent representation of abdominal adipose tissue who seek a versatile technique that allows for potential urethral lengthening at a later stage. This option is suitable for those willing to accept limited neophallus sensitivity while prioritizing minimal donor site morbidity.

The results of our study confirmed those described in the original report^18^ and in a recent multicenter study presented by Falcone et al.,^5^ in terms of reliability and surgical safety.

A different approach was described by Terrier et al.^36^ in 2014, aiming to maximize the size of the neo‐phallus and reduce postoperative complications. In contrast to the technique used in our series, the technique described by Terrier et al. involved the construction of three stages of the neophallus, each 3 months apart, followed by glans shaping and implantation of penile prostheses:

1 Implantation of two subcutaneous tissue expanders, subsequently inflated to distend the abdominal skin
2 Tabularization of the expanded skin paddle
3 Liberation of the upper extremity of the flap to create the neophallus, which maintains its natural connections with the pubic area

The multistage reconstruction led to results comparable to our series, in terms of both length and circumference of the neo‐phallus. The higher incidence of postoperative vascular complications in our sample (12.5% vs. 2.8%—percentage including partial and total flap necrosis), is balanced by a higher rate of general complications, especially due to the multiple steps required (99% vs. 27.5%, combined percentual of minor and major complications).^53^ Despite the advantage of multistage reconstruction in reducing abdominal scar, the multiple surgeries required to perform it may increase patient morbidity and anxiety and represent an additional financial burden. Therefore, we prefer to perform the first step of SPP in a single stage, as described in the original report.

### 4.1 Urethral lengthening

One of the main disadvantages of an SPP compared with a RAFFF is the lack of an incorporated functional neourethra. In patients who do not desire to urinate from the tip of the phallus or who have multiple cardiovascular risk factors, a simple burial of the clitoris with scrotoplasty can be performed, leaving the vagina and the female orthotopic meatus in place, as mentioned before.^32^

On the other hand, if patients desire to have a urethral reconstruction, various techniques can be taken into account:

1) Staged RAU is the preferred technique in our center. First described by Garaffa et al.,^20^ this procedure is based on the construction of the neourethra from the previously described radial fascio‐cutaneous flap and its incorporation into the neophallus. This is followed by the proximal urethral join‐up, performed in one or two steps, generally associated with ablation vaginectomy and the creation of the posterior urethra through an inverted flap from the anterior wall of the vagina. The minimal donor site morbidity reported with this technique is associated with a low incidence of urethral complications, with fistula formation in two (7%) and strictures in one (5%) of 27 patients. An overall flap loss rate of 7% was reported in this series. In order to minimize vascular complications, careful selection must be carried out prior to proceeding with the neo‐phallus construction.
2) Perineal urethrostomy can be configured using a urethral advancement with the mobilization of an anterior wall vaginal flap, followed by an ablation vaginectomy, burying of the clitoris, and scrotoplasty. In our opinion, this option represents an excellent choice for patients not requiring a micturition in orthostatism as it allows the creation of a male perineum while minimizing postoperative complications. Currently, no data are available in the literature to get to valuable conclusions.
3) Combined labia minora flap and skin graft reconstruction as described by Bettocchi et al.^18^ in the original article. This technique was burdened by a massive incidence of complications, 64% and 55% incidence of fistulas and stenosis respectively.
4) Metoidioplasty with urethral lengthening^30^, ^44^, ^54^ can be performed when the previous options are not viable alternatives for patients. In this case, the clitoris and the urethra will not be incorporated into the resulting penoid after SPP, with the external urethral meatus positioned at the base of the neophallus. This is not a standard procedure, and the discussion of urethral outcomes falls beyond the scope of this article.

SPP is, therefore, a safe and versatile technique that can be tailored to the individual needs and characteristics of each patient. Although this approach requires multiple surgical procedures—potentially causing distress—and the combination of different types of flaps, these aspects appear to offer certain advantages. Notably, the typical 3‐month interval between the first and second surgical stages allows for flap stabilization through vascular autonomization.^26^, ^27^, ^55^ Although the optimal interval for SPP or other phalloplasty techniques has not yet been established, the reported autonomization period for free flaps in the literature varies from 1 to 12 weeks,^27^ a timeframe that is therefore met. Once this process is complete, the flap becomes stable and is not at risk during subsequent procedures. Finally, in line with recent international trends, a gradual, multistep approach utilizing different types of flaps minimizes donor‐site morbidity while maintaining a complication rate comparable to that of free flaps.^22^, ^23^, ^24^, ^28^

Although an effective and safe total urethral reconstruction can be offered to patients undergoing SPP, in our series only slightly more than one‐third desired it, in particular, two patients required a metoidioplasty, eight a perineostomy, whereas an RAU was performed in the remainders four. This further underlines the contrast with the criteria listed by Hage and Gilbert, confirming that the patient's priority should be considered when choosing the type of genital reconstruction, including urethroplasty, in accordance with the standard of care of the WPATH criteria,^17^ offering a variety of surgical options, weighing the advantages and limitations of each technique and searching with the patient for the best suitable alternative.

### 4.2 Aesthetical outcomes

In terms of aesthetic outcomes, and when urethral lengthening is not required, SPP offers the advantage of eliminating the need for an additional donor site, while the residual abdominal scar can be easily concealed in daily life. Conversely, in cases requiring neourethral reconstruction, additional scarring at the donor site may occur. While this is a drawback, it remains less noticeable and less extensive than the scarring associated with a free radial flap, as previously mentioned.^22^, ^23^, ^24^ Finally, it should be emphasized that the shape of the neofallus is not cylindrical but pyramidal, tending to widen at the base and that, being dorsally positioned, the flap scar is not in the ideal position, thus being permanently visible and potentially becoming a disturbing element.

### 4.3 Erogenous outcomes

The main limitation of SPP remains the lack of erogenous sensitivity in the phallus. Tactile sensitivity is present but very limited, being mainly maintained at the base of the neophallus and absent at the tip. While the clitoris remains available for stimulation, other reconstructive techniques allow for better sensation and orgasmic function through phallic stimulation.

### 4.4 Risk factors and main complications

The known risk factors for postoperative complications were shown to have no impact on the rate of postoperative complications in our series. This may be due to the low sample size, however, it should be considered that we are analyzing a technique based on the use of a pedicled flap, requiring microanastomosis only in cases of RAU (4 out of 40 patients in our series) and generally tending to be less prone to vascular risk factors than other techniques based on the use of free flaps.^56^

#### 4.4.1 Smoking

Despite the absence of complications associated with vascular flaps in our series, smoking is a well‐documented risk factor for vascular disease. Studies have demonstrated that active smokers are at a higher risk of developing complications following free flap reconstruction than nonsmokers.^57^ Ischemic complications in free flap phalloplasty can range from 5 to 10%,^58^ with the potential for complete phallic necrosis. For this reason, in our center, it is recommended that patients quit smoking at least 6 months before the surgery.

In a court of patients unwilling to quit smoking, a combined SPP plus RAU technique is not recommended. In these cases, the alternatives are to proceed with one of the techniques previously described or to forgo urethral lengthening.

#### 4.4.2 Body mass index

Patients with a BMI > 30 are generally considered less ideal candidates for flap reconstruction due to the significantly increased risk of postoperative complications, particularly vascular issues. Therefore, in our center, a dietary consultation prior to surgery is recommended. Despite this, SPP remains a good technique for patients with a sufficient amount of abdominal adipose tissue. We discourage the use of SPP in underweight patients (BMI < 18), as the phallus size would be limited, and the closure of the abdominal defect could present challenges. On the other hand, we do not have a strict BMI cutoff for obese patients, as the flap can be easily defatted up to Scarpa's fascia and tabularized in a tension‐free manner, yielding good results. Additionally, abdominal defect closure in these patients can often be performed directly, without the need for rotational flaps.

#### 4.4.3 Diabetes mellitus

Diabetes mellitus remains one of the main risk factors for the development of microvascular complications and surgical wound infections.^59^, ^60^ Strict control of diabetes before surgery is recommended in accordance with international standards.^61^ In our patient cohort, diabetes was diagnosed in only one case, and no association with postoperative complications was observed. Although there is no strict cut‐off, a glycosylated hemoglobin level above 8.5% constitutes an exclusion factor for performing microvascular techniques.

#### 4.4.4 Vascular complications

Being supplied by its own vascular pedicle, this flap does not require microanastomosis, reducing operative time and the risk of vascular complications. In cases of ischemia, typically confined to the distal part of the neophallus, an outpatient surgical procedure involving debridement and segmental suturing can be easily performed, resulting in an acceptable residual neophallus length.

In our series, surgical debridement was necessary in 10.5% of cases, while a secondary TPC was required in one case due to total phallic loss. Although the incidence of vascular complications may seem high, most are minor, while major complications—such as arterial or venous thrombosis—are rare.

For instance, in the case of RAFFF, reported postoperative vascular complication rates include approximately 15.8% for partial necrosis of the neophallus,^8^, ^62^ 1.7–6.2% for acute arterial thrombosis,^4^, ^63^ and 1.8–5.9% for acute venous thrombosis.^8^, ^62^ Rates of surgical revision and total penile loss are reported at 10.8% and 1.8%, respectively.^64^

In general, SPP‐based TPC remains a viable alternative with a reduced risk of vascular complications. A positive impact on reducing vascular complications could be achieved by modifying the surgical technique—for example, by incorporating the perforating branches of the deep inferior epigastric vein—or by using advanced diagnostic technologies, such as indocyanine green fluorescence^25^ or forward‐looking infrared tomography.^65^

However, these modifications come with limitations. While they may improve perfusion, they would also significantly increase surgical time, ultimately compromising the key advantages of this technique: simplicity and minimal operative duration. The aforementioned diagnostic technologies could indeed help prevent and reduce vascular complications. However, they are not available at our center, and their cost‐effectiveness remains to be demonstrated.

Despite RAFFF being our reference option for GGAS, the aforementioned issues demonstrate that the SPP may be an effective alternative for certain patients who are current smokers or overweight/obese.

The absence of a comparative group or randomization the limited follow‐up and the lack of patient‐reported outcomes analysis represent the main limitations of our study.

## 5 CONCLUSION

Our evidence suggests that SPP is a reliable and technically accessible option for GGAS in transgender men, particularly when microsurgery is contraindicated. The technique offers the advantage of accommodating the patient's needs for urethral lengthening, simplifying the surgical process, and reducing operative times. While vascular complications may occur relatively frequently, they are mostly minor and can be managed with simple outpatient procedures.

In the absence of evidence supporting the superiority of one technique over another, our results suggest that SPP is a reliable option for dealing with TPC in the transgender male population.

## AUTHOR CONTRIBUTIONS

Marco Flacone designed the study; Lorenzo Cirigliano and Mirko Preto carried out the data acquisition and the first revision of the manuscript. Mattia Anfosso analyzed the data and drafted the manuscript. The manuscript was then critically revised by Marco Falcone and Paolo Gontero. All authors approved the submitted and final version.

## CONFLICT OF INTEREST STATEMENT

The authors have no conflict of interest to disclose.
